# Linking Gene Expression and Functional Network Data in Human Heart Failure

**DOI:** 10.1371/journal.pone.0001347

**Published:** 2007-12-19

**Authors:** Anyela Camargo, Francisco Azuaje

**Affiliations:** School of Computing and Mathematics, University of Ulster at Jordanstown, Newtownabbey, Northern Ireland, United Kingdom; Center for Genomic Regulation, Spain

## Abstract

**Background:**

Gene expression profiling and the analysis of protein-protein interaction (PPI) networks may support the identification of disease bio-markers and potential drug targets. Thus, a step forward in the development of systems approaches to medicine is the integrative analysis of these data sources in specific pathological conditions. We report such an integrative bioinformatics analysis in human heart failure (HF). A global PPI network in HF was assembled, which by itself represents a useful compendium of the current status of human HF-relevant interactions. This provided the basis for the analysis of interaction connectivity patterns in relation to a HF gene expression data set.

**Results:**

Relationships between the significance of the differentiation of gene expression and connectivity degrees in the PPI network were established. In addition, relationships between gene co-expression and PPI network connectivity were analysed. Highly-connected proteins are not necessarily encoded by genes significantly differentially expressed. Genes that are not significantly differentially expressed may encode proteins that exhibit diverse network connectivity patterns. Furthermore, genes that were not defined as significantly differentially expressed may encode proteins with many interacting partners. Genes encoding network hubs may exhibit weak co-expression with the genes encoding their interacting protein partners. We also found that hubs and superhubs display a significant diversity of co-expression patterns in comparison to peripheral nodes. Gene Ontology (GO) analysis established that highly-connected proteins are likely to be engaged in higher level GO biological process terms, while low-connectivity proteins tend to be engaged in more specific disease-related processes.

**Conclusion:**

This investigation supports the hypothesis that the integrative analysis of differential gene expression and PPI network analysis may facilitate a better understanding of functional roles and the identification of potential drug targets in human heart failure.

## Introduction

Heart failure (HF) stems from complex genetic, environmental and life style factors and is one of the main causes of death in the world [1]. Myocardial infarction, ischemic cardiomyopathy and dilated cardiomyopathy (DCM) may contribute to the emergence of HF. The latter, is a leading cause of congestive heart failure [1], [2]. In DCM, the heart becomes enlarged, which makes the pumping of blood less efficient to vital organs. Due to the high rate of morbidity and mortality attributed to HF, previous studies have aimed to unveil the genetic factors crucial to the emergence and development of the disease. As a result, HF signature genes [3], [4], [5], protein-protein interactions (PPI) and larger gene expression data sets have been made publicly available [6]. Thus, it has been suggested that the integration of these sources may improve the identification of clinically-relevant disease markers [6], [7].

Recent examples of the predictive power of integrative bioinformatics approaches to investigating diseases have been reported by Oti et al. (2006) [8] and Xu and Li (2006) [9]. They investigated whether signature genes of genetically heterogeneous and hereditary diseases could be predicted from the analysis of PPI networks. Lu et al. (2007) [10] integrated gene expression analysis and a biological interaction network to investigate the allergic response in asthma. Cline et al. (2007) [11] proposed a generic protocol to integrate gene expression data and biological networks, which may help to explain the control mechanisms underlying the observed changes in activity of a biological process. In the context of HF, Barth et al. (2006) [2] analysed gene expression patterns related to DCM and identified specific gene regulatory relationships. Here, using a DCM-related microarray data set, we report an analysis of human HF gene expression responses in relation to a HF-specific PPI network.

To build the HF PPI network, known HF-relevant genes (KHFG) were first identified together with validated PPIs of their encoded proteins according to the Human Protein Reference Database (HPRD) [12]. This was followed by the identification of differentially expressed genes from microarray data encoding molecular profiles of healthy vs. HF subjects. The proteins encoded by these significantly differentially-expressed genes were mapped onto the global HF PPI network. We first assessed key statistical and topological relationships between significantly differentially-expressed genes and the interaction network. Results showed that in terms of gene expression, genes significantly differentially expressed are not always represented by highly-connected nodes. Other results showed that, although not significantly differentially-regulated, some of the proteins encoded by genes traditionally associated with HF may interact with proteins encoded by significantly differentially-expressed genes, and that the latter tend to be highly connected. Further analyses, which integrated expression data and the PPI network, evaluated levels of co-expression between genes encoding network nodes and corresponding genes encoding their interacting partners. A key question was whether there was any significant quantitative relation between co-expression levels and network connectivity degree. This analysis indicate that: a) genes represented by network hubs may exhibit weak co-expression with the genes encoding their interacting protein partners; and b) genes that were not defined as significantly differentially-expressed in the gene expression data analysis may encode proteins with many interacting partners. These significant findings were replicated using a second, independent microarray data set. To identify biological process overrepresentations associated with the PPI network's topology, the data were analysed in the context of the Gene Ontology. Results show that genes represented by highly-connected nodes are more likely to be engaged in higher-level biological process terms (as defined by the Gene Ontology), than genes represented by low-connectivity nodes. This study supports the idea that a PPI network integrated with expression data may further assist researchers in identifying potential disease markers or therapeutic targets, which might be overlooked when results rely on expression profiling analyses only.

## Results

This study evaluated human HF gene expression responses, in relation to the topology of a HF-specific PPI network. The microarray data set analysed was obtained from the Gene Expression Omnibus (GEO) [13], accession number GDS2206. This data set, which was derived from a study on DCM, consisted of 28 samples: 15 and 13 samples obtained from non-failing hearts and HF patients respectively. After pre-processing, significantly differentially-expressed genes were identified by performing significance analysis of microarray (SAM) [14]. To validate significant findings, a second microarray data set was used. This second data set, also derived from a study on DCM, was obtained from the GEO, accession number GDS2206, and included 12 samples: 5 samples originated from non-failing hearts and 7 samples from HF patients (see Methods).

The PPI network was assembled by including validated interactions, as reported in the HPRD [12], for KHFGs and for proteins encoded by genes included in the expression data sets. For the network visualisation, a colour labelling scheme was used to distinguish between the types of proteins each node represented. In addition, nodes were classified according to the degree of connectivity. Superhubs were represented by nodes with connectivity degree greater than 100, hubs referred to nodes with connectivity degree greater than 20 and lower than 100, peripheral-A were nodes with connectivity greater than two and lower than 20; and peripheral-B nodes represented proteins with one interacting partner (see Methods).

To evaluate relationships between connectivity and significantly differentially-expressed expression patterns, topological analysis of the network was carried out. Furthermore, network topology was integrated with the DCM expression data to evaluate gene connectivity versus co-expression levels. To calculate co-expression levels linked to every node in the network, the nodes and their interaction partners were mapped into the DCM expression data set. Next, Pearson's correlation coefficients between the expression profile of each gene coding for a node and each gene coding for its interaction partners were calculated. Correlation value pairs were regarded as significantly co-expressed if P-value<0.01. Finally, the co-expression level of a gene was calculated by comparing the number of its significantly co-expressed interactions against the total number of its interacting proteins (see Methods). The PPI network was analysed in the context of Gene Ontology (GO) to identify biological process overrepresentations. Over-represented biological processes were ranked according to their position in the GO hierarchical scheme [15] (see Methods).

### Network analysis

**Figure 1:**
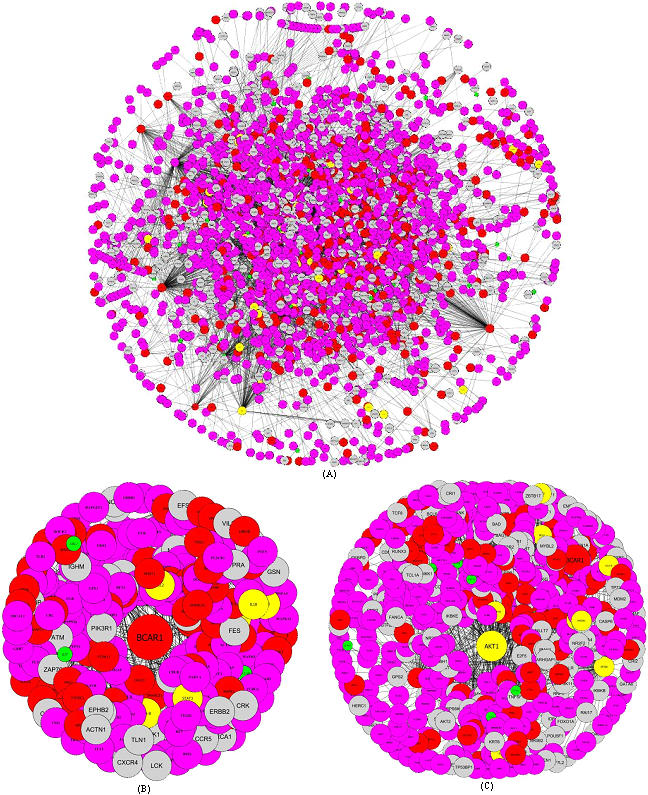
PPIs networks. PPIs networks corresponding to (A) global human HF network, (B) BCAR1's PPI network, (C) AKT1's PPI network. All PPIs were retrieved from the HPRD. Up-regulated genes are represented by red nodes. Down-regulated genes are represented by green nodes. Known HF genes (KHFG) are represented by nodes in yellow. Other genes encoding interacting partner proteins are represented by nodes in purple, if they have a corresponding transcript, or in grey if they have no corresponding transcripts in the gene expression data set.

The PPI network (Figure 1A) consisted of nodes representing proteins and their interaction partners. Some of the nodes represented proteins encoded by significantly differentially-expressed genes obtained from expression pattern analysis. Initially, 1161 genes were identified (974 up-regulated in DCM and 187 down-regulated in DCM). However, only 506 (457 up-regulated and 49 down-regulated genes) were represented in the network because their encoded proteins were reported to have at least one interacting protein partner. The network also contained 71 nodes representing proteins encoded by KHFGs only. The network contained 2835 nodes representing proteins encoded by not significantly differentially-expressed genes (Table 1).

**Table 1:** Summary of nodes population according to connectivity.

Hierarchy	SEg	KHFG	N-SEg	Total %
Superhub	4	3	0	0.21
Hub	43	24	5	2.11
Peripheral-A	279	37	585	26.41
Peripheral-B	180	7	2245	71.28
Total	506	71	2835	3412

Summary of nodes population according to connectivity. % nodes within each category present in the interaction network. Significantly differentially-expressed genes (SEg). Non-significantly differentially-expressed genes (N-SEg). Known HF genes (NHFG).

According to the node hierarchy described in Methods, 2.3% of the genes were represented by network hubs or superhubs corresponding to 47 significantly differentially-expressed genes (4 superhubs and 43 hubs). In contrast, 97.0% of the genes were represented by either network peripheral-A or peripheral–B nodes corresponding to 459 significantly differentially-expressed genes (279 peripheral-A and 180 peripheral-B). Details are shown in Table 1. Three statistical significance tests based on random sampling were implemented to allow us to reject the null hypothesis that the proportion of significantly differentially-expressed genes (which were also categorised as either hubs or superhubs) was obtained by chance. These tests are described in Methods. All the statistical significance tests reported *P* = 0. Thus, this supports the conclusion that the observed proportions are statistically significant, i.e. larger than the proportions expected by chance.

Examples of potentially relevant associations are described as follows. *BCAR1* is a protein represented by a network superhub (Figure 1B) and *NAP1L1* is represented by a peripheral-B. According to the gene expression analysis, both *BCAR1* and *NAP1L1* encoded significantly differentially-expressed genes. *AKT1* also represents a network superhub (Figure 1C). The gene encoding *AKT1* is known to be associated with HF and it is involved in several functional processes relevant to the development of this disease, such as Apoptosis and the *MAPK* (Mitogen-activated protein kinase) signalling pathway [16].

Only 40, out 71, KHFGs had a corresponding transcript in the DCM data set, and only one of these genes, *SOD1*, was significantly differentially-expressed. Moreover, 2051 genes that encoded other protein's interaction partners in the network had a corresponding transcript in the gene expression data set and were not significantly differentially-expressed. There were KHFGs with no corresponding transcripts in the DCM data set because these were either included in the array but with significant missing values across the experimental samples, or their probes were not included in the array.

### Network connectivity versus significant gene expression patterns

**Figure 2:**
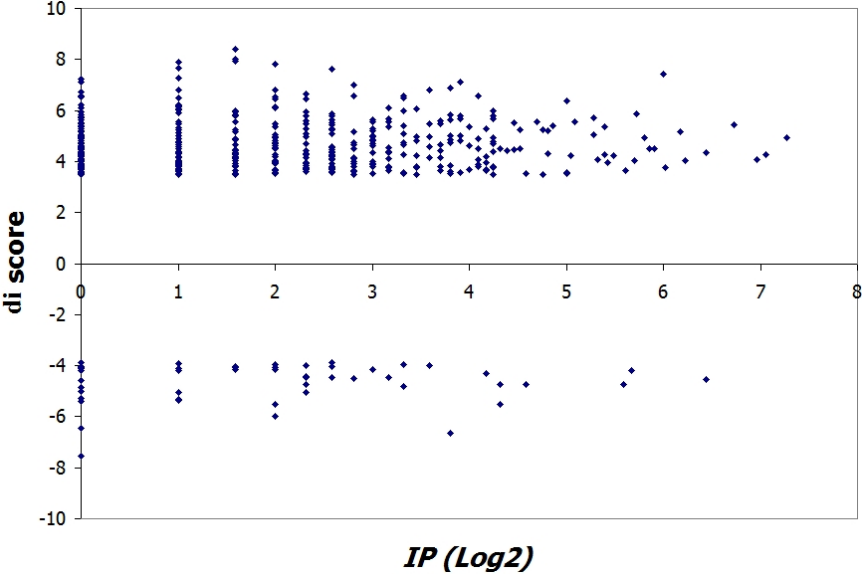
Plot of t-statistics (*cL*). Plot of t-statistic (*di*) representing the score for gene *i* vs. number of interacting partners (IP Log2) associated with protein encoded by gene *i*.

This section of the study integrated gene expression data with the PPI network to describe potential significant relationships between network connectivity and gene expression patterns (as described in Methods). The first set of results, involving significantly differentially- expressed genes, found that genes represented by network superhubs and hubs tend to have lower range of ‘*di*’s values (the score of class differentiation). In Figure 2 genes with those characteristics are shown on the farthest right side of the plot. On the contrary, genes represented by network peripherals-A and -B tend to have higher range of ‘*di*’s values. When proteins encoded by non-significantly differentially-expressed genes were assessed, we found that some of these protein's interacting partners were encoded by several significantly differentially-expressed genes. For instance, *GRB2* has 180 interacting partners and was not found to be significantly differentially-expressed in the gene expression data. However, 19 genes encoding its interacting partners, such as *ABL1* or *BCAR1*, were identified as significantly differentially-expressed in the expression data analysis. We analysed the biological role of *GRB2*, and its corresponding interacting partners, and found that processes such as ‘signal transduction’, “regulation T Cell activation” or “regulation of *MAPK* activity” were over-represented (P<0.0001). According to KEGG and Reactome, *GRB2* is involved in more than 15 pathways. Other proteins whose interacting partners were encoded by more than 15 significantly differentially-expressed genes were *TP53*, *NR3C1*, *SMAD2*, *CASP3*, *ESR1*, *RB1* and *YWHAG* (Annex S1 shows complete list), which are involved in functional processes such as apoptosis or cell cycle. These findings stress the importance of performing gene expression analysis in conjunction with interaction networks to help to identify otherwise overlooked potential clinically-relevant targets.

### Gene co-expression analyses in the context of network connectivity

**Figure 3:**
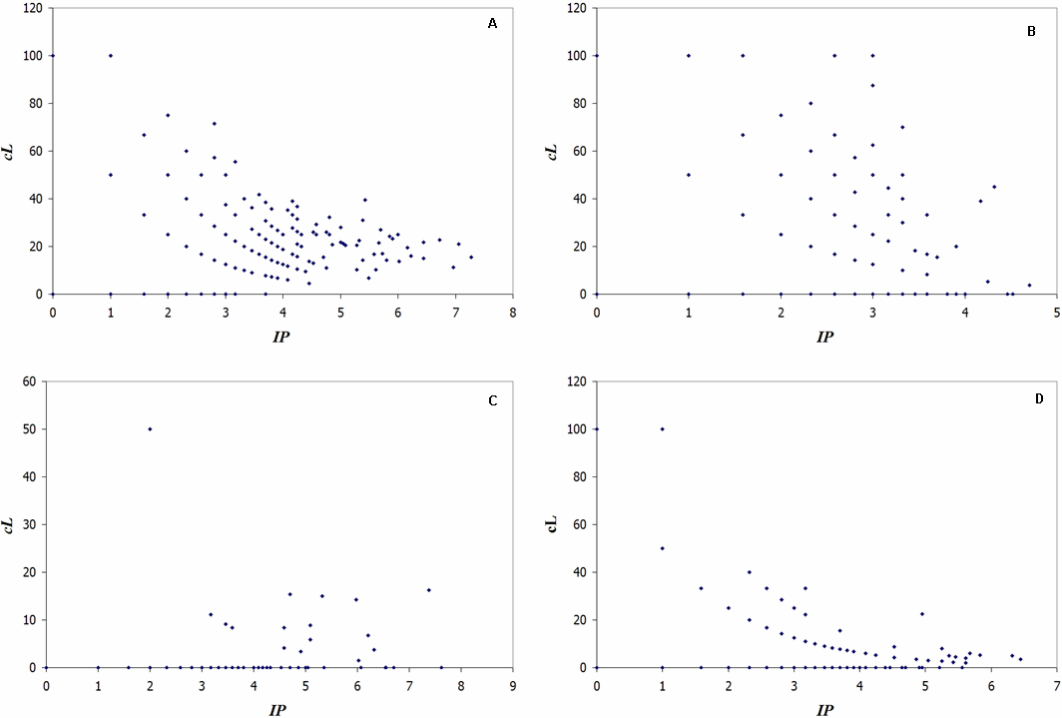
Scatter plot of the number interacting partners (*IP_i_*) for a gene *i*, versus its significant co-expression level (*cL_i_*). (A) significantly differentially-expressed genes. (B) Non-significantly differentially-expressed genes. (C) KHFGs. (D) Independent testing data set.

In this section gene expression data were integrated with the topology of the PPI network to assess significant *co-expression levels* (as detailed in Methods). We found that genes represented by network hubs and superhubs are not necessarily significantly co-expressed with their attributed protein-coding partners (IPs), than other types of nodes. For example, genes MAPK1 and FXR2, represented by network superhubs, were significantly co-expressed with 15.5% and 10.2% of the genes encoding their IPs respectively. On the other hand, genes represented by network peripheral-A and -B may be strongly correlated with their interacting partners. For example, ALDOB, represented by a network peripheral-A, was significantly co-expressed with all the genes encoding its IPs (i.e. 100% significant co-expression level). Table 2 shows more details of the difference between these categories in terms of mean *cL* values. No statistical significance difference between category means were found at *P* = 0.05. However, note that only network peripherals-A or –B showed cases with *cL* = 100%. The global trend, as shown in Figure 3, is that the higher the number of node connections the greater the tendency to display low *cL* values. Figure 3A shows a scatter plot of the number interacting partners (*IP_i_*) for a gene *i*, versus its significant co-expression level (*cL_i_*) (as defined in Methods). Similar trend was observed when non-significantly differentially expressed genes were analysed. For example, *HAP1* and *SIN3A*, represented by network peripherals-A, were significantly co-expressed with all their partners, *IPs*. Figure 3B plots (*IP_i_*) versus (*cL_i_*) of non-significantly differentially-expressed genes. When analysing nodes representing KHFGs, results showed that in general *cL_i_* of these genes was low. In fact, none of the 40 KHFGs, which had a corresponding transcript in the DCM expression data, obtained a *cL_i_*>50% (Figure 3C). For example, the *cL_i_* for *PRKCA*, represented by a network superhub, was equal to 16.2% (i.e. this gene's expression pattern was significantly co-expressed with only a few of the genes coding for its *IPs*).

**Table 2:** Summary of average co-expression levels (*cL*) for each network node category.

Categories	Average *cL* ± SD
Superhubs	17.33 ± 4.59
Hubs	15.45 ± 10.39
Peripherals-A	16.51 ± 26.65
Peripherals-B	14.48 ± 35.21

Summary of average co-expression levels (cL) for each network node category. SD: Standard deviation. No statistical significant difference between category means were found at *P* = 0.05.

In order to estimate the potential biological relevance of the significant relationships found, as well as their reproducibility, these pattern association procedures were replicated on a second, independent human HF gene expression data set. Figure 3D, which reviews the overall results of this second analysis, corroborates the findings presented above. Genes encoding network hubs and superhubs tend to be more weakly co-expressed with the genes coding for their network interacting partners (*IP_i_*) than those genes represented by network peripherals-A and –B. For example, *cL_i_* of genes encoding network superhubs and hubs, such as *AR*, *MAPK1*, *PTPN11* or *RAF1*, were lower than 5%. On the contrary, the *cL_i_* of *MRFAP1L1*, encoding a network peripheral-A, was equal to 100%.

The final analysis of this sequence evaluated the variation of co-expression levels (*cLs*) across the four categories of network nodes. Gene's *cL* values were grouped according to node category and the significance of the variance among these categories was estimated by *F*-statistics. Results indicated (Table 3) that there are significant differences between high connectivity categories (hubs and superhubs) and low connectivity categories (peripherals-A and –B) (*F* = 10.93, *P*<0.000001). By contrast, *F*-statistics found no significant difference when comparing high connectivity categories (network hubs vs. superhubs) (*F* = 4.53, *P* = 0.14). Further, analysis of variance (ANOVA) among all four categories was performed, from which no significant differences were found (*F* = 0.93, *P* = 0.42). This analysis suggests that hubs and superhubs display a significant diversity of co-expression patterns in comparison to peripheral nodes.

**Table 3:** Analysis of variance of co-expression levels (*cL*) across categories of network nodes.

Categories compared	*F*-value	*P*-value
Hubs vs. superhubs	4.53	0.14
Hub vs. peripheral-A	7.41	0
Hub vs. peripheral-B	12.99	0
Superhub vs. peripheral-A	33.66	0.003
Superhub vs. peripheral-B	58.92	0.001
Peripheral-A vs. peripheral-B	1.75	0
Superhub, Hub vs. peripheral-A,-B	10.93	0
ANOVA (between all categories)	0.93	0.42

Analysis of variance of co-expression levels (cL) across categories of network nodes. Significance level from *F*-statistics is represented in the form of *P*-values.

Genes were ranked according to their co-expression levels (*cL*), such that a gene, *i*, was defined as *low co-expressed* if its *cL_i_* was below 0.80 and *highly co-expressed* if its *cL_i_* was greater or equal than 0.80. For example, *MAPK1*'s *cL* is equal to 0.15, therefore the gene was labelled as low co-expressed. An example of a highly co-expressed gene is *ALDOB*, whose *cL* is equal to 1.0 (100% significant co-expression). Once the genes were grouped according to this criterion, F-statistics were applied to estimate variability between significant co-expression levels of these two groups. We found that the difference of significant co-expression levels between these two groups was statistically significant (*F* = 103.97, *P*<0.00001). Through this analysis, we found that the *cLs* of *MAP3K5* and *PABPC1* (represented by network peripherals-B) were of 0.80 and 0.87 respectively. Watanabe and Otsu (2004) [17] reported that *MAP3K5* promotes heart dysfunction and dilation, as well as cardiac fibrosis. A previous study by Deo et al. (2002) [18] linked *PABPC1* to *SOD1*, the latter was the only KHFG significantly differentially-expressed in our expression analysis.

### GO analysis

The PPI network topology was also assessed in the context of the GO to identify major biological roles within each node category. Genes represented by network peripherals-A tend to be involved in a greater number of lower-level GO biological processes (e.g. “CD4-positive, alpha-beta T cell differentiation during immune response”, “G1/S transition of mitotic cell cycle”, “insulin-like growth factor receptor signalling pathway”) than those represented by network hubs and peripherals-B (Table 4). The “G1/S transition of mitotic cell cycle” biological process has been reported to be fundamental in the development of HF [3]. Table 4 also shows that genes represented by network superhubs tend to be involved in a greater number of higher-level biological processes (e.g. “cell communication” or “signal transduction”). These results are consistent with results obtained by Lu et al. (2007) [10] in an asthma model. However, we also demonstrated that genes represented by network peripherals-B tend to perform higher-level biological processes (Annex S2 contains complete list of over-represented GO biological processes). But the latter may be explained by the relative lack of experimental studies of these genes.

**Table 4:** GO analysis, significantly over-represented biological processes.

Category	% higher-level GO terms	% lower-level GO terms
Superhub	0.29	0.71
Hub	0.15	0.85
Peripheral-A	0.06	0.94
Peripheral-B	0.15	0.85

Significance level <0.05.

This analysis also corroborates that some genes found to be not significantly differentially-expressed may influence the activation or repression of several other gene products. For example, the protein encoded by *NR3C1* gene is known to interact with 82 proteins. GO analysis of *NR3C1*, and corresponding interacting partners, identified more than 60 significantly over-represented biological processes. Examples of such relevant functional categories are “transcription” or “transcription from RNA polymerase II promoter” (P<0.00001). This gene was not significantly differentially-expressed, but its encoded protein interacts with 17 proteins encoded by genes that were significantly differentially-expressed in the expression data analysis (e.g. *HNRPU*, *MAPK1*, *PRPF6*, *RAF1*, *NFKB1* and *STAT3*). These genes are known to be involved in crucial processes relating to cardiac remodelling, such as apoptosis, *MAPK* signalling pathway or immune system signalling as described in KEGG and Reactome databases and by other authors [3], [16], [19]. In addition, Kang and Izumo (2000) [16] suggested that DCM is a one of the most common forms of HF associated with apoptosis pathways dysfunction.

## Discussion

Undoubtedly gene expression pattern analyses have provided insights into the biological basis of several deadly diseases. Because of this, an important question is how to take advantage of available public data and information bases. Moreover, there are concerns about the reproducibility of functional predictions and pattern identification results obtained using different data sets and platforms.

In human heart failure, a fair amount of microarray expression data have been produced and uploaded into public databases. Moreover, a number of disease signature genes have been reported [2], [3], [7]. The challenge now is to find ways to integrate such information in order to facilitate the discovery of disease-specific knowledge and targets, as well as their reproducibility using different data sources. This study evaluated gene expression responses from the perspective of a PPI network and several external functional information sources. Quantitative analysis approaches were applied to elucidate significant global patterns and trends encoded in these data sources. In particular, we address the following questions: a) how network-based targets relate to significantly differentially (or not differentially) gene expression-based targets; and b) how network connectivity relates to significant levels of co-expression between interacting partners. To answer these questions a PPI network based on proteins encoding KHFGs and other expressed genes was built. Significant quantitative relationships between gene expression data and the PPI network were identified. Such relationships and relevant functional patterns may allow one to identify significant genes and specific biological processes, which may be overlooked when analyses rely on gene expression data only. Furthermore, significant quantitative relationships were reproduced using an independent data set.

The integration of expression data and the PPI network allowed us to identify functionally-important, influential genes, which were not found to be significantly differentially expressed in the expression data analysis. Some of the proteins encoded by these genes were reported to influence relatively large sets of proteins, which may be encoded by significantly differentially-expressed genes. For instance, proteins encoded by genes *SMAD2*, *CASP3*, *ESR1* and *RB1*, interacted with 17, 16, 16 and 15 proteins encoded by significantly differentially-expressed genes. In the case of KHFGs, nearly 50% of them had no corresponding transcripts in the DCM data sets. However, the proteins they encoded were reported to interact with several others proteins, which were encoded by significantly differentially-expressed genes. For example, the protein encoded by *STAT3* (represented by a network hub) was reported to interact with other 94 proteins. Among them, nine were encoded by significantly differentially-expressed genes, such as *MAPK1* or *PDIA3*, which are involved in functional pathways such as natural killer cell mediated cytotoxicity or MAPK signalling. These pathways are involved in the aetiology of HF [3]. In addition, evidence based on published papers has demonstrated the involvement of *STAT3* in the protection of the myocardium from HF [20], [21].

The integrative analysis of gene expression data and the PPI network suggests that proteins represented by network peripherals-A and -B tend to be encoded by genes that are significantly co-expressed with most of the genes encoding their interacting proteins. This relationship was not observed in the case of network hubs and superhubs. In terms of biological significance, these analyses also showed that genes encoding network superhubs and hubs tend to be involved in higher-level biological processes as defined by the GO Biological Process hierarchy. Furthermore, genes represented by network peripherals-A tend to be associated with lower-level biological processes.

The integrative study reported here highlights three important aspects. First, the approaches implemented may help to identify potentially influential genes (e.g. *STAT3*, *TGFB1*, *AKT1*, *SIN3A*, *PABPC1*, *MAP3K5*), otherwise overlooked by single-source expression pattern analysis. Second, it also represents a powerful methodology to trace biological processes that may outline potential clinical biomarkers or therapeutic targets and their corresponding interacting partners. Third, in combination with expression data this approach can be used as a tool to evaluate stimulus/response studies. For example, two microarray data sets can be mapped onto an interaction network to analyse gene repression or activation when different experimental conditions are studied. The latter application will be reported as part of a forthcoming study.

Despite the fact that the global human HF network presented here is far from complete and that it may include false positive interactions, biologically-significant quantitative and qualitative relations were identified. Moreover, significant quantitative findings were reproduced when an independent gene expression data set was analysed. Quantifiable differences between potentially influential functional components (as predicted by independent single-source analyses and categories) were also found [2], [3], [7].

A key conclusion of this research is that only a minority of hubs (or superhubs) genes are also significantly differentially expressed in HF. Furthermore, we showed that the observed proportions of such genes are statistically significant in comparison to the values expected by chance. The biological relevance of these results can be summarised as follows. First of all, this study confirms the weakness of performing functional characterisations of genes based on gene expression data only. The integrative analysis of gene expression and functional network data may improve the predictive ability of future studies. The identification of potential drug targets in heart failure should include integrative approaches to estimate significant roles of genes in regulatory processes driving the emergence of heart failure. Second, this study suggests that processes relevant to cardiac remodelling and the progression toward heart failure may be controlled by greater gene expression modifications of low-connectivity network nodes, and relatively smaller differential responses in nodes with higher connectivity. Moreover, hubs and superhubs display a significant diversity of quantitative co-expression patterns in comparison to peripheral nodes. This may suggest that gene expression coordination between hubs (or superhubs) and their interacting partners may be more subtle that that observed between other genes.

## Materials and Methods

### Microarray data analysis

The microarray data analysed in this study were obtained from the Gene Expression Omnibus GEO) [13], accession number GDS2206. This data set, which was derived from a study on DCM, was composed of 28 samples: 15 and 13 samples from non-failing hearts and HF patients respectively [2], [22]. This data set was available in Log scale [2], and probes with missing values in more than 50% of samples, in either group, were excluded. To quantity differential gene expression, significance analysis of microarray (SAM) [14] was performed. The algorithm computes a *t* statistic, *di*, representing the score of class differentiation for gene *i*. Gene expression differences were considered significant if False Discovery Rate (FDR) <0.05 and Folding change >1.2.

In addition, a second microarray data set was used to validate significant findings (i.e. gene co-expression vs. network patterns) obtained from the first gene expression data set. This second data set, also derived from a study on DCM, was obtained from the GEO, accession number GDS2206, and was composed of 12 samples: 5 samples originated from non-failing hearts and 7 samples were obtained from HF patients [2], [22].

### A human HF interaction network

A PPI network was assembled by including validated interactions reported for KHFGs and for proteins encoded by genes included in the gene expression data sets. This network is offered as a public resource of the current status of human HF-relevant interactions (network is provided on request). The list of KHFG was obtained from the Entrez database [23]. Entrez query was restricted by the same set of keywords used in King et al. (2003) [3] (i.e. smooth muscle, endothelial cell, apoptosis, cytokine and adhesion molecule) and within the context of human HF. PPIs were retrieved from the HPRD [4]. The HUGO nomenclature standard was used to define unique id identifiers.

The PPI network was assembled by using a routine written in JAVA, and its structure was encoded in the SIF format that can be used by well-known network visualisation tools (e.g. Cytoscape). The product of this assembly was a network composed of 3412 nodes and 13164 interactions. The number of interacting partners range from 1 to more than 100. A colour labelling scheme was used to distinguish between the types of proteins each node represented. Proteins encoded by up- and down-regulated genes (as predicted in the gene expression data) were represented by nodes coloured in green and red respectively. Proteins encoded by KHFGs were represented by yellow nodes. Proteins encoded by not significantly differentially expressed genes were represented by purple nodes. Proteins encoded by genes whose expression pattern was not present in the data set, but which encoded relevant interacting partners in the HF network, were represented by grey nodes.

Nodes in the network were also classified according to the degree of connectivity, based on a scheme similar to that used in Lu et al. [10]. *Superhubs* are represented by nodes with connectivity degree greater than 100, *hubs* refer to nodes with connectivity degree greater than 20 and lower than 100, *peripheral-A* are nodes with connectivity greater than two and lower than 20; and *peripheral-B* nodes represent proteins with one interacting partner only.

Cytoscape v2.4 [24] was used for network visualisation.

### Comparing observed vs. expected proportions of relevant nodes

The statistical significance of the observed proportion (*R_obs_*) of significantly differentially-expressed genes that were also either hubs or superhubs was estimated by three independent statistical significance tests. In these analyses the null hypothesis was that the observed proportion was obtained by chance, i.e. a random null distribution of genes could have reported proportions as extreme as the value observed. These statistical significance analyses were implemented as follows. Based on the results shown above, each gene was assigned to one of the following labels: “Significantly differentially-expressed” (SDE) or “not-SDE”. This vector of labels will be referred to as “expression labels”. Similarly, each gene was labelled according to the binary class: “Superhub-or-hub” or otherwise. This vector of labels will be referred to as “connectivity labels”. The first statistical significance assessment was based on a permutation test as follows. The expression labels were randomly shuffled to obtain a permutated dataset. Using this dataset, the proportion (*R_per_*) of SDE that were also either superhubs or hubs was calculated. This permutation process was repeated *N* times, and the number of times, *numSigPer*, that *R_per_≥R_obs_* was calculated, with *R_obs_* = 47/3412. Thus, the statistical significance of the observed value (probability that the observed value was obtained by chance) was estimated by *P* = *numSigPer*/*N*, with *N* = 50000. A second permutation test was conducted in a similar fashion, but now with only the connectivity labels randomly shuffled to generate the permutated datasets. In the third significance assessment test, 506 proteins (i.e. the number of SDE genes observed) were randomly sampled from the total population of network nodes (3412 proteins). Based on this sample, *R_rand_* represents the proportion of genes that were both SDE and that encoded either hubs or superhubs. This sampling process was repeated *M* times, and the number of times, *numSigRand*, that *R_rand_*≥*R_obs_* was calculated, with *R_obs_* = 47/506. Thus, the probability of finding *R_obs_* at random was estimated by *P* = *numSigRand*/*M*, with *M* = 50000. All the statistical significance tests reported *P* = 0, with maximum *R_per_* and *R_rand_* values equal to 20/3412 and 19/506 respectively.

### Network vs. gene co-expression analysis

Results from the topological analysis of the network were integrated with the results obtained from the gene expression data analysis in order to evaluate relationships between connectivity and significantly differentially expressed expression patterns. Furthermore, network topology was integrated with the DCM expression data to evaluate gene connectivity versus co-expression levels.

To calculate co-expression levels linked to every node (*i*) in the network, node and their interaction partners were mapped into the gene expression data set. Next, Pearson's correlation coefficients between the expression profile of each gene coding for a node and each gene coding for its interaction partners were calculated. Correlation value pairs were regarded as significantly co-expressed if its P-value<0.01. Finally, the *co-expression level* (*cL*) of a gene (*i*) was calculated by comparing the number of its significantly co-expressed interactions (*cIP_i_*) against the total number of its interacting proteins (*IP_i_*), i.e. significantly co-expressed and non-significantly co-expressed interaction partners (Eq. 1). According to Eq. 1, a co-expression level (*cL_i_*) equal to 100% indicates that the gene (*i*) was significantly co-expressed with each of the genes encoding its interacting partners in the PPI network. A co-expression level (*cL_i_*) equal to 0% indicates that the gene (*i*) was not significantly co-expressed with any of its interacting partners.
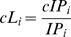
Assuming that a node, *i*, in the PPI network represents a protein encoded by a gene, then: *i* is a node in the network; *IP_i_* represents the set of interacting partners linked to *i*; *cIP_i_* represents the set of interacting partners significantly co-expressed with *i*.

To verify that major findings were reproducible, the same procedures were carried out using a second, independent microarray data set as described above.

### Gene Ontology analysis

Interaction network topology was analysed in the context of the GO. Cytoscape-BiNGO [25] was applied to detect significantly over-represented GO biological processes. Benjamini and Hochberg multiple-test corrections adjusted raw *P*-values at a significant level<0.05. To increase the level of stringency, GO-IEA terms were discarded. GO annotations with IEA evidence code refer to annotations inferred from sequence-based similarity searches, which have not been reviewed by curators. In addition, using the GO interaction network, a hierarchical classification scheme was used to rank over-represented GO biological processes according to their proximity to the root node of the GO Biological Process hierarchy. Note that the GO root node is at the top level of the hierarchy, followed by the Biological Process term and the rest of the hierarchy terms (e.g., “developmental process” (third level), “multicellular organismal development”, on the fourth level). In this study a *higher-level Biological Process* is defined as any term above the fourth level in the GO Biological Process hierarchy. A *lower-level Biological Process* is defined as any annotation subsumed by any term in the fourth level in the GO Biological Process hierarchy.

## Supporting Information

Annex S1 Network proteins and corresponding number of interacting partners encoded by significantly differentiated genes
(0.17 MB XLS)

Annex S2 Significantly over-represented GO biological processes.
(0.09 MB XLS)
